# Oral anticoagulation in elderly patients as secondary prevention of cardioembolic strokes

**DOI:** 10.1186/1755-7682-3-8

**Published:** 2010-06-05

**Authors:** Lorena Benavente, Sergio Calleja, Vanessa de la Vega, Jorge García, Carlos H Lahoz

**Affiliations:** 1 Department of Neurology, Hospital Universitario Central de Asturias, Spain

## Abstract

**Background:**

Stroke incidence increases with age. Atrial fibrillation (AF) is an important risk factor for ischemic stroke and its incidence also increases with age. However oral anticoagulant therapy (OAT) tends to be underused in the elderly population.

**Methods:**

Elderly patients (> = 80 years) with an ischemic stroke admitted in our department between 1/7/2003 and 31/6/2005 were prospectively evaluated. Baseline characteristics, risk factors, treatment and etiology according to TOAST criteria were recorded. Patients treated with OAT were followed up in order to assess any side effect and stroke recurrence. Mean follow-up was of 19.5 months (7-45) from discharge.

**Results:**

Sixty four out of a hundred and fifty nine elderly patients (40.25%) were classified as cardioembolic; mean age was 84.5 years (80-97) and 64.6% were women. AF had been previously identified in 60% of them (16.9% were on OAT and 40.6% on antiplatelet therapy). At discharge, 32 patients (49.2%) were on OAT. In the follow-up 4 patients (12.5%) suffered systemic haemorrhages (3 urinary, 1 gastrointestinal bleeding), with no change in their functional status. Mean INR in this group was 5.9 [3-11] and, in 3 of them, OAT was cancelled. No brain haemorrhages were recorded. Ischemic stroke recurred in 4 patients (INR < 1.8 in 3 of them; the other, INR 2.35). Three patients had died at the end of the follow-up, one of them as a consequence of ischemic stroke recurrence.

**Discussion:**

Twenty eight point eight of stroke patients admitted in the period of study were >80 years. The high proportion of cardioembolic strokes in this age segment contrasts with the general underuse of OAT as antithrombotic prophylaxis. Our study suggests that OAT is a safe strategy when carefully prescribed, even for elderly patients.

## Introduction

Atrial fibrillation (AF) is present in more than 5% of >65 year-old population, increasing with age, as it is present in 10% of people ≥ 75 years [1]. Cardioembolism is the etiology in approximately 25% of strokes in patients age ≥ 80 years [2]. It is also known that paroxistic AF, very common in elderly people, raises the same risk of stroke as chronic AF [3]. Non-valvular atrial fibrillation (NVAF) is the most common cause of arrythmia, as the frequency of reumathic valvulopathy falls [1]. AF predisposes to left atrial thrombus formation and carries a sixfold increased anual risk for stroke, rising from 1.5% for those aged 50-59 years to 23.5% for those aged 80-89 years [1], [3]. Such cardioembolic strokes are very often more severe than other ischemic strokes, and are associated to a higher mortality [4], [5], [6]. The annual embolic rate attributed to AF is approximately 12% for patients >65 years-old [7], [8], [9]. It has been shown that the NIHSS and modified-Rankin scales, patients on oral anticoagulation therapy show less serious strokes and present a better functional situation at discharge than those who were not on that treatment [10]. This seems to endure as a controversial subject that leads physicians to a difficult decision when they prescribe these patients antithrombotic profilaxis.

The main objective or our study was to prospectively assess the efficacy and safety or oral anticoagulants (OA) in ≥ 80 year-old population as secondary prophylaxis of cardiembolic strokes.

## Subjects and Methods

This is an observational prospective study conducted in tertiary hospital that covers an area of 400,000 habitants and it is the reference hospital of a department of one million habitants. To be eligible, patients had to be ≥ 80 years of age and a diagnosis of ischemic stroke or transient ischemic attack (TIA) had to be present. According to these inclusion criteria 159 patients were consecutively selected among all the stroke patients admitted in our Neurology Department starting July 1^st ^2003 until June 31^st ^2005. We analysed clinical characteristics, cardiovascular risk factors, previous treatment and at discharge treatment and follow-up, but also classified the stroke etiology according to TOAST criteria [11]. Patients treated with oral anticoagulants were followed up through periodical reviews, the first one at our Outpatient Clinic, 3 months from discharge; after that we have been in contact with the patients by phone every 6 months.

At admission in the Emergency Department, a brain computerized tomography (CT), blood ancillary tests including hemogram, glucose, electrolytes, vitamins, renal, liver and thyroid function, lipids, coagulation and INR (International Normalized Ratio) if having oral anticoagulants; electrocardiogram and thoracic radiography were performed in all patients.

Demographic data were recorded for all 159 patients, including age and sex, social status, previous treatment and treatment at discharge, previous stroke incidence (TIA, brain infarction or haemorrhagic brain infarction) or other neurological diseases. Other cardiovascular risk factors were also studied, including previous diagnoses of hypertension (BP ≥ 140/90), diabetes (basal glycaemia ≥ 110 mg/dl), dyslipemia (total cholesterol ≥ 220 mg/dl or LDL-cholesterol ≥ 160 mg/dl), ischemic cardiopathy, myocardiopathy, peripheral artery disease, smoking (one of more cigarettes per day) or alcohol intake (≥40 g/day). The severity of stroke and functional outcome were reflected by NIHSS, Barthel and Modified-Rankin scales.

The etiological classification has been led according to TOAST criteria [11] after practising ancillary studies, such as carotid ultrasonographic study, transcranial Doppler, Holter monitoring, echocardiogram and other specific blood analyses on an individual basis. Therefore, the strokes were classified as "cardioembolic strokes", "large-artery atherosclerosis", "small-artery occlusion", "stroke of other determined etiology" and "stroke of undetermined etiology".

We selected those patients diagnosed with cardioembolic stroke according to TOAST classification, summarizing 64 patients (42.1%) out of the total of the sample. According to the social status, risk of falls and mainly neurological and functional scales, we treated 32 of them (40.9%) with oral anticoagulants, avoiding this treatment in case of patients without social support, high risk of falls or m-Rankin score over 3. We have followed them up, analysing the adverse events related to these drugs and the clinical outcome.

## Results

Five hundred and thirty patients were admitted in our Department with a diagnosis of ischemic stroke. One hundred and fifty nine of them (30%) were more than 80 years old and were selected for our study. Sixty five per cent were females, thirty five per cent were males, and mean age was 84.5 years. According to TOAST criteria, cardioembolic strokes represented 40.25% (64) of our patients, followed by large-artery atherosclerosis and small-artery occlusion representing 4.1% of our patients each. Fifty one point fifty five per cent were undetermined, twenty per cent of them for coexisting different etiologies. All the patients underwent electrocardiogram and carotid ultrasonography; holter monitoring was practised in 23% of patients and Echocardiography in 11.6% of patients.

### Cardioembolic stroke population

**Figure 1:**
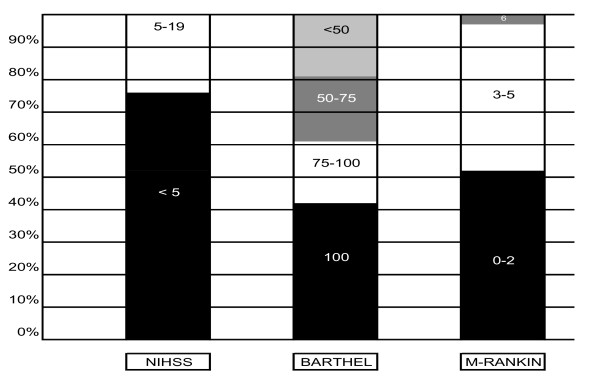
**Clinical and dependency scales at discharge**.

Looking at the cardioembolic subgroup, the clinical diagnosis was transient ischemic attack (TIA) in 7.8%, brain infarction in 90.6%, and haemorrhagic infarction in 1.6%. Cardiovascular risk factors (CVRF), previous treatments and neurological status at discharge are shown in Table 1, and clinical and dependency scales at discharge are also represented in Figure 1. Only 27% of patients with previously known AF were on anticoagulant therapy.

**Table 1:** Clinical characteristics of cardioembolic stroke patients.

CARDIOVASCULAR RISK FACTORS	
HT	107 (67.2%)
AF	92 (57.8%)
PREVIOUS STROKE	56 (35.1)
DIABETES	35 (21.9%)
ISCHEMIC CARDIOPATHY	30 (18.8%)
DYSLIPEMIA	21 (13.2%)
MYOCARDIOPATHY	15 (9.5%)
PERIPHERAL ARTERY DISEASE	5 (3.1%)
PREVIOUS TREATMENTS	
ANTIHYPERTENSIVES	94 (59.4%)
STATINS	23 (14.3%)
ANTIPLATELETS	66 (41.3% )
ANTICOAGULANTS	25 (15.6%) (27% OF KNOWN AF)

Cardiovascular risk factors, previous treatments, dependency scales at discharge.

### Cardioembolic stroke patients treated with oral anticoagulant

**Figure 2:**
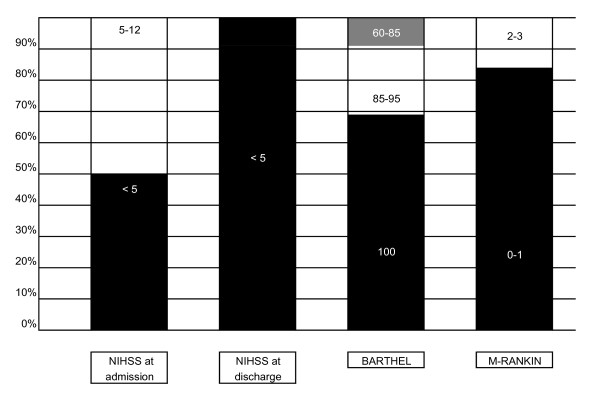
**Clinical and dependency scales at discharge**.

Out of all cardioembolic stroke patients, we treated with OA 40.9% of them (n = 32). Gender, age, CVRF, social support and clinical and dependency situation at discharge are showed in Table 2, and clinical and dependency scales at discharge are also represented in Figure 2. Patients on OA therapy were followed for a mean of 19.5 months (7-45) and the adverse events related to oral anticoagulation were as follow: Four patients (12.5%) suffered from a systemic haemorrhage: 3 had urinary bleeding and 1 had a gastrointestinal haemorrhage, although the M-Rankin scale score was not modified in any case. The mean INR in these patients was 5.95 [3-11]. On the other hand ischemic recurrence affected another 12.5% (4 patients) whose INR was less than 1.8 in three of them and an optimized level (INR = 2.35) in the other one. Three patients died during the follow-up because of myocardial infarction, pneumonia and recurrent ischemic stroke respectively. Withdrawal of treatment occurred in three cases, one because of a gastrointestinal haemorrhage, and the other two following the recommendations of primary healthcare physicians. There was not any intracranial haemorrhage.

**Table 2:** Clinical characteristics of cardioembolic stroke patients treated with oral anticoagulant.

GENDER AND AGE	
SEX	40.6% male
AGE	83.2 (80-89)
CARDIOVASCULAR RISK FACTORS	
HT	21 (65.6%)
AF	21 (65.6%)
DIABETES	7 (21.9%)
DYSLIPEMIA	6 (19.4%)
ISCHEMIC CARDIOPATHY	5 (15.7%)
MIOCARDIOPATHY	4 (12.9%)
CONGESTIVE CARDIOPATHY FAILURE	4 (12.9%)
SYSTEMIC EMBOLISM EVENTS	2 (6.3%)
PERIPHERAL ARTERY DISEASE	1 (3.1%)
SOCIAL SUPPORT	
LIVING WITH FAMILY	27 (84.4%)
LIVING ALONE	4 (12.5%)
INSTITUTIONALIZED	1 (3.1%)

## Discussion

It has been previously demonstrated that AF is a powerful risk factor for stroke. Our results show that AF is very frequent among elderly stroke patients, rising cardioembolic stroke etiology to 40.25%. Oral anticoagulants have been proven to reduce the risk of stroke in AF by 68% compared with placebo, which is superior to the relative risk reduction of 21% associated with full-dose aspirin [12], [13], [14], [10], [15], [16]. Even more, a recently published study has suggested that aspirin at 150-200 mg per day is neither effective nor safe [17]. Despite the modest or even dubious effect associated to aspirin, this drug is widely used in antithrombotic atrial fibrillation prophylaxis as in spite of its proven benefits, numerous studies have documented an underuse of warfarin, particularly among elderly patients who would seem to benefit the most from anticoagulant therapy [18], [19]. However, OA as prophylactic treatment are fourfold increased in people younger than 80 years [17]. Haemorrhages, falls, and patient refusal or a history of nonadherence to therapy constituted nearly 80% of the physician-cited reasons for not prescribing warfarin to elderly patients [20], [21]. Other studies conclude that patients at high risk for falls with atrial fibrillation are at substantially increased risk of intracranial haemorrhage, especially traumatic haemorrhage; nevertheless, because of their high stroke rate, they appear to benefit from anticoagulant therapy if they have multiple stroke risk factors [22], [23], [24], [25]. Moreover it has been suggested that oral anticoagulation treatment not only prevents stroke but may also contribute to the development of less serious strokes with a better functional prognosis [16], [26], [25].

In our study, among 64 patients identified with AF, 40.9% (n = 32) were discharged on oral anticoagulants, selected according to the neurological and dependency scales and excluding other contraindications, as current evidence-based guidelines suggest [27], [28]. Throughout-the follow-up of these patients (7-45 months), OAT was shown as an efficient and safe treatment in these selected patients, despite being a very old population (80-89 years-old). With respect to haemorrhage risk, systemic haemorrhages were found in only four cases, without modifying the M-Rankin scale, and they corresponded to overtreated patients, as the INR reflected (mean 5.95). We did not find any intracranial haemorrhage. Although atrial fibrillation was present as a risk factor in more than a half of cardioembolic strokes, only a quarter of these patients were previously an adequate anticoagulant prophylaxis.

In summary, we would like to remark the importance of cardioembolic etiology in strokes of patients over 80 years, which contrasts with the scarcity in the appropriate use of OAT as prophylaxis. According to our results, stroke recurrence in patients on oral anticoagulation is mainly ischemic, and haemorrhagic complications that derive from oral anticoagulation would be related to overdosing. Having into account that the population is too small and the follow-up relatively short, this paper suggests that OAT might be safe in cardioembolism prophylaxis independently of age and may be effective if we compare with natural history of not anticoagulated patients [13], [14], [16], [20].

## Competing interests

The authors declare that they have no competing interests.

## Authors' contributions

LB carried out the search of data and patients management, drafted the manuscript and participated in the design of the study and performed the statistical analysis. SC conceived of the study and participated in its design and coordination, and also carried out patients' management and follow-up. VV and JG contributed to the data collection. CHL participated in the coordination. All authors read and approved the final manuscript.
